# Comparison of the Unfolded Protein Response in Cellobiose Utilization of Recombinant Angel- and W303-1A-Derived Yeast Expressing β-Glucosidase

**DOI:** 10.3389/fbioe.2022.837720

**Published:** 2022-03-31

**Authors:** Shaolan Zou, Yudie Jia, Qing He, Kun Zhang, Rui Ban, Jiefang Hong, Minhua Zhang

**Affiliations:** ^1^ Tianjin R&D Center for Petrochemical Technology, Tianjin University, Tianjin, China; ^2^ School of Chemical Engineering and Technology, Tianjin University, Tianjin, China; ^3^ Key Laboratory for Green Chemical Technology of Ministry of Education, Tianjin University, Tianjin, China; ^4^ School of Pharmaceutical Science and Technology, Tianjin University, Tianjin, China

**Keywords:** recombinant yeast, UPRE-*lac*Z, β-glucosidase, displaying and secreting, cellobiose, ethanol and acid, UPR response, metabolic burden

## Abstract

The unfolded protein response (UPR) is one of the most important protein quality control mechanisms in cells. At least, three factors are predicted to activate the UPR in yeast cells during fermentation. Using UPRE-*lac*Z as a reporter, we constructed two indicator strains, KZ and WZ, based on Angel-derived K-a and W303-1A strains, respectively, and investigated their UPR response to tunicamycin, ethanol, and acetic acid. Then, four strains carrying plasmids BG-cwp2 and BG were obtained to realize the displaying and secretion of β-glucosidase, respectively. The results of cellobiose utilization assays indicated interactions between the UPR and the metabolic burden between the strain source, anchoring moiety, oxygen supply, and cellobiose concentration. Meanwhile, as expected, growth (OD_600_), β-glucosidase, and β-galactosidase activities were shown to have a positive inter-relationship, in which the values of the KZ-derived strains were far lower than those of the WZ-derived strains. Additionally, extra metabolic burden by displaying over secreting was also much more serious in strain KZ than in strain WZ. The maximum ethanol titer of the four strains (KZ (BG-cwp2), KZ (BG), WZ (BG-cwp2), and WZ (BG)) in oxygen-limited 10% cellobiose fermentation was 3.173, 5.307, 5.495, and 5.486% (v/v), respectively, and the acetic acid titer ranged from 0.038 to 0.060% (v/v). The corresponding maximum values of the ratio of β-galactosidase activity to that of the control were 3.30, 5.29, 6.45, and 8.72, respectively. Under aerobic conditions with 2% cellobiose, those values were 3.79, 4.97, 6.99, and 7.67, respectively. A comparison of the results implied that β-glucosidase expression durably induced the UPR, and the effect of ethanol and acetic acid depended on the titer produced. Further study is necessary to identify ethanol- or acid-specific target gene expression. Taken together, our results indicated that the host strain W303-1A is a better secretory protein producer, and the first step to modify strain K-a for cellulosic ethanol fermentation would be to relieve the bottleneck of UPR capacity. The results of the present study will help to identify candidate host strains and optimize expression and fermentation by quantifying UPR induction.

## 1 Introduction

*Saccharomyces cerevisiae* is one of the most widely used cell factories and is utilized in biotechnological processes including the production of heterologous proteins, biofuels, and chemicals of medical or industrial interest (Jouhten et al., 2016; Lian et al., 2018; Thak et al., 2020). In synthetic biology and metabolic engineering applications, yeast not only faces many kinds of stress, such as osmotic, heat, inhibitor, and nutrient starvation stress, but also endures a metabolic burden resulting from the heterologous production of enzymes to broaden the yeast’s substrate or product range (Mattanovich et al., 2004; Wu et al., 2016; Brandt et al., 2021).

Protein synthesis, folding, and processing are tightly controlled and are sensitive to perturbation of endoplasmic reticulum (ER) homeostasis in all organisms. The unfolded protein response (UPR) is a conserved intracellular signaling pathway that regulates the transcription of ER homoeostasis-related genes (Dallbey et al., 2009; Young and Robinson, 2014). *S. cerevisiae* is a major model for studying UPR mechanisms. Research has focused on protein quality control pathways and mechanisms using endogenous or exogenous protein expression in yeast (Dallbey et al., 2009; Young and Robinson, 2014; Cedras et al., 2019; Sun and Brodsky, 2019). However, in recent years, other factors have been reported to induce the UPR (Navarro-Tapia et al., 2016; Kawazoe et al., 2017; Navarro-Tapia et al., 2018). Ethanol stress has been shown to alter membrane fluidity, which then activates the UPR; thus, ethanol tolerance might be improved by enhancing the UPR (Navarro-Tapia et al., 2016; Navarro-Tapia et al., 2018). Acetic acid has also been demonstrated to cause ER stress and lead to UPR (Kawazoe et al., 2017).

In fact, the UPR is so important that it is increasingly becoming a limiting factor in the exploitation of yeast. Quantification of UPR induction and modulation of the UPR and ER-Associated Degradation (ERAD) activity is increasingly applied in yeast synthetic biology and metabolic engineering (Cedras et al., 2019; Thak et al., 2020). However, current knowledge about the UPR pathway and its mechanism in yeast is inadequate, especially the regulatory role of the UPR underlying stress adaptation, the metabolic burden, and their mutual interaction.

In the bio-energy field, although *S. cerevisiae* is the preferred microorganism in various biofuel production configurations, its application in second-generation (2G) fermentation involving lignocellulose conversion remains challenging (Lian et al., 2018; Brandt et al., 2021). One of the key difficulties is the low titer of cellulase expression, which is reported to possibly be limited by the UPR or ERAD or both (Davison et al., 2020). Furthermore, because ethanol and acetic acid are constantly present, either as a target in the bio-energy field or as the main by-product in other application fields (Navarro-Tapia et al., 2016; Kawazoe et al., 2017; Navarro-Tapia et al., 2018), the role of ethanol and acetic acid and their mutual interaction with other activators in the UPR are also important.

β-Glucosidase is an essential and key component in lignocellulose bio-conversion and is also an important enzyme in medicine and industry (Bhatia et al., 2002; Wang et al., 2013; Ding et al., 2018). The expression of β-glucosidase in yeast leads to enzyme production or the production of whole cell biocatalysts for cellulosic ethanol fermentation (Wang et al., 2013; Ding et al., 2018; Cedras et al., 2019; Davison et al., 2020). The haploid strain K-a is derived from the diploid industrial yeast strain TH-AADY (Angel Yeast Co., Ltd., Yichang, China) and has been proven to have good ethanol fermentative performance and stress resistance. Thus, in this study, the enzyme BGLI (β-glucosidase) from *Aspergillus aculeatus* was selected as a reporter protein to investigate the effects on the UPR of genetically different host strains (industrial K-a and a laboratory strain W303-1A), heterologous enzyme expression, displaying or secreting mode, and fermentation conditions. Then, the unfolded protein response element (UPRE)-*lac*Z was used as a reporter gene to evaluate the UPR (Amberg and Huo, 2009; Dallbey et al., 2009). In the present study, the UPRE was a hybrid promoter and contained a 22-base pair sequences, which allows Hac1p (a UPR-associated transcription factor) binding to activate the UPR (Dallbey et al., 2009). Assays to measure β-galactosidase activity were performed to determine the UPR induction levels (Amberg and Huo, 2009). In addition, 2 and 10% cellobiose were used to observe the possible effects from metabolic products, including ethanol and acetic acid. The relative expression of several key target genes was further investigated. The results of this study are expected to assist in identifying candidate host strains and for optimizing the expression mode and fermentation condition by quantifying UPR induction.

## 2 Materials and Methods

### 2.1 Strains, Plasmids, Media, and Growth Conditions

The microbial strains and plasmids used in this study are listed in Table 1 (Wang et al., 2013; Ding et al., 2018; Lu et al., 2019; Zhang et al., 2020). *Escherichia coli* Top10 was used for recombinant DNA manipulation. Recombinant plasmids were constructed and amplified in Top10 cultivated at 37°C in a Luria-Bertani liquid medium or on Luria-Bertani agar (1% tryptone, 1% NaCl and 0.5% yeast extract, pH7.0). Ampicillin was used at a final concentration of 100 g/L. WZ, that was W303-1A (*leu* 2::UPRE-*lac*Z) (Zhang et al., 2020), was constructed by integrating the donor DNA fragment UPRE-*lac*Z into gene *leu* 2 site with the CRISPR-Cas9 method (Lu et al., 2019). The haploid yeast strain K-a, obtained by the sporulation of the commercial Angel yeast strain TH-AADY (diploid, Alcohol active dry yeast, Angel Yeast Co., Ltd., Yichang, China, http://www.angelyeast.com) and then counter selection on a 5′-FOA plate, was used as the host to express exogenous cellulases (Lu et al., 2019; Zou et al., 2021). Yeast strains were generally cultivated at 30°C in rich YPD or YPC medium (1% yeast extract, 2% peptone, 2% glucose or cellobiose), or basal CMG or CMC medium (6.7 g/L yeast nitrogen base without amino acids, 20 g/L glucose or cellobiose, and the appropriate amino acid and nucleic acid supplements) (Wang et al., 2013).

**TABLE 1:** Microbial strains, plasmids, and primers used in this study.

Strains/plasmids/primers	Essential properties	Source or reference
*Escherichia coli* Top10	F^−^ *mcr*A (*mrr*-*hsd* RMS-*mcr*BC)80 *lac*ZM15 *lac*X74 *rec*A1 *ara*139 (*ara*-*leu*)7,697 *gal*U *gal*K *rps*L (Str^R^) *end*A1 *nup*G	In our lab
*S. cerevisiae*	*MAT*a *ade*2 *trp*1 *his*3 *can*1 *ura*3 *leu*2	In our lab
W303-1A		
WZ	W303-1A (*leu* 2:: UPRE-*lac*Z)	In our lab
WZ (YEplac195)	Strain WZ containing plasmid YEplac195	In our lab
K-a	*MAT*a *ura*3, derivative from the diploid industrial yeast strains TH-AADY (Angel Yeast, Yichang, China)	In our lab
K-a (YCplac33-Cas9)	Strain K-a containing plasmid YCplac33-Cas9	In our lab
WZ (BG)	Strain WZ containing plasmid YEplac195-P*tpi*-*xyn*2s-Aa BGL1-T*adh*I	In this study
WZ (BG-cwp2)	Strain WZ containing plasmid YEplac195-P*tpi*-*xyn*2s-Aa BGL1-*cwp*2-T*adh*I	In this study
KZ	K-a (*leu* 2::UPRE-*lac*Z)	In this study
KZ (YEplac195)	Strain KZ containing plasmid YEplac195	In this study
KZ (BG)	KZ containing plasmid YEplac195-P*tpi*-*xyn*2s-Aa BGL1- T*adh*I	In this study
KZ (BG-cwp2)	KZ containing plasmid YEplac195-P*tpi*-*xyn*2s-Aa BGL1- *cwp*2-T*adh*I	In this study
YCplac33-Cas9	*Amp^r^*, *URA 3*, Cas 9, 5,603 bp (shown in Figure 1 and Supplementary Material Data Sheet 2)	In our lab
pRS42H-gRNA	*Amp^r^*, *hph N*T1 crRNA	In our lab
pRS42H-gLEU2	*Amp^r^*, *hph N*T1, crRNA, 20 bp guide for *LEU* 2 gene (shown in Figure 1 and Supplementary Material Data Sheet 1)	In our lab
YEplac195	*Amp^r^*, *URA*3	In our lab
BG	YEplac195-P*tpi*-*xyn*2s-Aa BGL1-T*adh*I, β-glucosidase secreting expressing vector	In our lab Wang et al. (2013)
BG-*cwp*2	YEplac195-P*tpi*-*xyn*2s-Aa BGL1-*cwp*2-T*adh*I, β- glucosidase displaying expressing vector, containing 207 bp *cwp*2 sequence encoding an anchored peptide to display the expressed β-glucosidase on the cell surface	In our lab Ding et al. (2018)
Primer P1	5′ CAC​AAT​TTG​CTA​AAG​GTA​CT 3′	Donor DNA synthesis
Primer P2	5′ CTT​GTG​ATT​CTT​TGC​ACT​TC 3′	
Primer P3	5′ TGA​CCA​AGT​TCG​TAA​ATC​TA 3′	Transformant identifying
Primer P4	5′ CCA​TCT​CCA​CAA​TAG​GCA​TA 3′	
HAC1-F	5′CTT​TGT​CGC​CCA​AGA​GTA​TGC​G3′	Product size 532/280 bp
HAC1-R	5′GTG​ATG​AAG​AAA​TCA​TTC​AAT​TCA​AAT​G3′	
ACT1-F	5′CAA​ACC​GCT​GCT​CAA​TCT​TC3′	Product size 150 bp
ACT1-R	5′AGT​TTG​GTC​AAT​ACC​GGC​AG3′	
IRE1-F	5′AAG​GCA​TCC​GTT​GTT​TTG​GC3′	Product size 128 bp
IRE1-R	5′AGT​CAG​AAC​CGG​CGT​CAA​AT 3′	
INO1-F	5′AGA​GAT​TGC​TCC​TTC​CAC​GA 3′	Product size 164 bp
INO1-R	5′ACT​TGG​TTT​GTC​CCG​ACT​TG 3′	
ERO1-F	5′TGA​AGG​AGG​CAG​GCA​AAT​CG 3′	Product size 150 bp
ERO1-R	5′TAC​CGT​TAG​AGG​GCC​TTG​GA 3′	
HLJ1-F	5′ATT​TGG​GCC​TTC​TGC​TTC​CA 3′	Product size 127 bp
HLJ1-R	5′TGC​TTG​TTG​TTG​CTG​CTG​TC 3′	
LHS1-F	5′GCT​CGT​CAG​GAG​TTG​CGT​AT 3′	Product size 149 bp
LHS1-R	5′AGT​AAA​AGC​CAA​ACG​GCT​GC 3′	
MPD1-F	5′CCC​CCA​ATG​AGG​GTC​CTT​TT 3′	Product size 109 bp
MPD1-R	5′TCG​TCG​TGC​TTG​TTT​CCT​GA 3′	
KAR1-F	5′ATT​CCA​CCA​GCA​CCA​AGA​GG 3′	product size 85 bp
KAR1-R	5′CTG​TGG​CAG​ACA​CCT​TCA​GA 3′	

### 2.2 DNA Manipulation, Plasmid Construction, and Yeast Transformation

**FIGURE 1:**
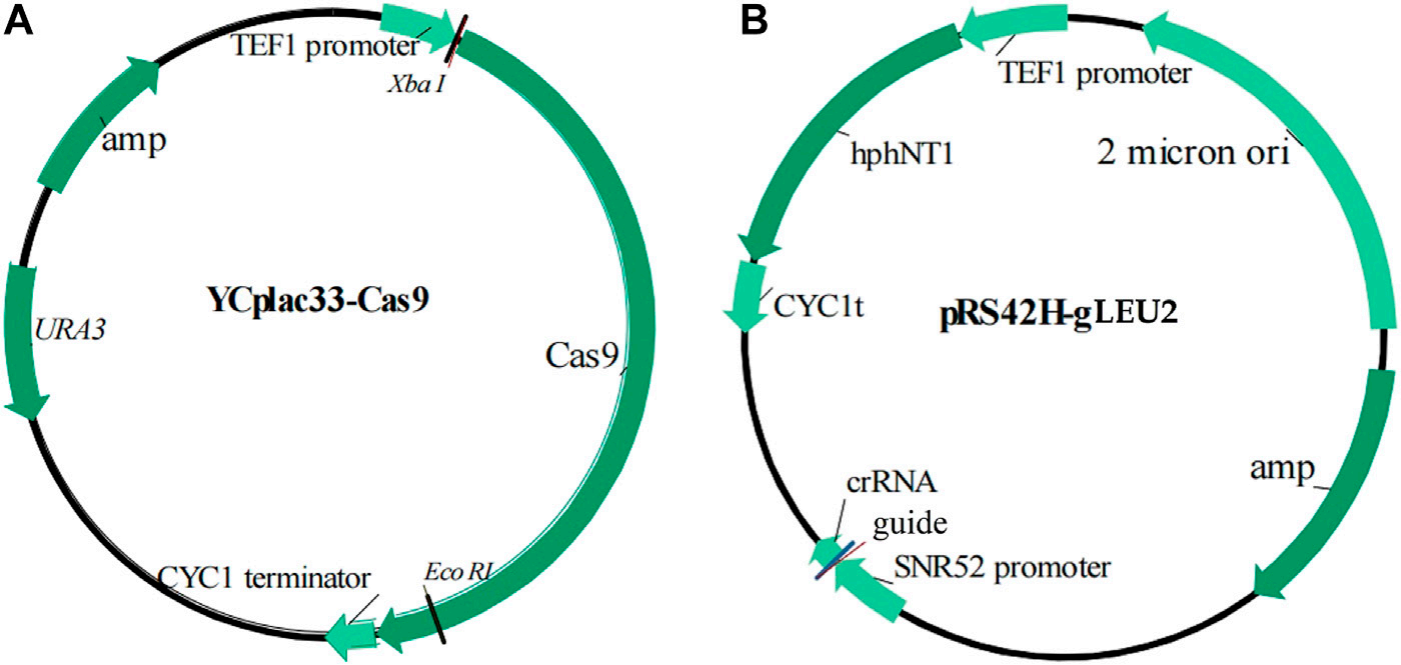
Schematic illustration of plasmids. **(A)** YCplac33-Cas9; **(B)** pRS42H-gLEU2.

Standard molecular genetic techniques were used for nucleic acid manipulations (Sambrook and Russell, 2001). The primers and plasmids used were listed in Figure 1, Table 1, and Supplementary Material.

The procedure to construct an indicating strain K-a (*leu 2*::UPRE-*lac*Z), also abbreviated as KZ, was shown as follows: 1) The primer pair P1/P2 was used to amplify 3,721 bp donor DNA fragment UPRE-*lac*Z by PCR with the genomic DNA of strain WZ as the template; 2) The aforementioned donor DNA and guide plasmid pRS42H-gLEU2 (Table 1) were co-transformed into the competent cells of strain K-a (YCplac33-Cas9) by the lithium acetate method (Gietz et al., 1995), and the screening plate used was CMG^−LEU^ with hygromycin B at the final concentration of 450 μg/mL; 3) The colonies grown on the aforementioned plates were copied to the plate YPD with tunicamycin and X-Gal (5-bromo-4-chloro-3-indolyl-beta-D-galacto-pyranoside). The blue colonies were further identified by PCR with genomic DNA as the template and the primers used were P3 and P4, which were all external to P1 and P2, respectively. The PCR products were predicted to be 4,327 and 795 bp for positive and negative colonies, respectively.

Plasmids YEplac195, BG, and BG-cwp2 (Table 1) were also transformed into the competent cells of the strains WZ and KZ by the lithium acetate method (Gietz et al., 1995). *URA3* was used as a selective marker and yeast transformants were screened in a CMG solid medium absent of uracil.

### 2.3 Growth and UPR Response Evaluation of Indicating Strains WZ and KZ in YPD With Different Additives

After being pre-cultivated twice in the YPD medium for 16–20 h at 30°C, the recombinant yeast indicator strains were grown aerobically to OD_600_ = 2.0 ± 0.1 in a fresh YPD medium with the initial OD_600_ value ∼0.2 at 30°C and 220 rpm. 50 ml of the resultant cultures were aliquoted into 250 ml shake flasks, mixed with different reagents, sealed with Parafilm films and then continued to grow at 30°C and 150 rpm. These cultures were sampled at regular time points for growth and UPR response analysis.

Growth analysis was generally conducted by detecting the optical density of the culture at 600 nm (OD_600_). UPR response analysis was carried out by β-galactosidase activity assay (Amberg and Huo, 2009). Protein content was measured with the Bradford protein assay kit according to the instruction book (Tiangen Biotech (Beijing) Co., Ltd.). One unit of activity (U) was defined as the amount of enzymes required to release 1 nmol of O- nitrophenol per minute under the assay condition.

### 2.4 Growth, β-Glucosidase and Ethanol Production, and UPR Response Evaluation of β-Glucosidase-Expressing Strains in Cellobiose

After being pre-cultivated in the CMG medium for 24 h, the recombinant yeast β-glucosidase-expressing strains were grown aerobically in a fresh medium for 24 h at 30°C. The resultant cells were collected by centrifugation, and these were washed twice with distilled water and then inoculated into 250 ml shake flasks containing 50 ml of rich media containing 2% or 10% cellobiose. The initial optical density at 600 nm (OD_600_) of the medium was adjusted to 0.2. These cultures were allowed to grow aerobically at 30°C with shaking at 220 rpm, or anaerobically by sealing with Parafilm films at 30 C with shaking at 150 rpm. Samples were collected at regular time points for growth, β-glucosidase or/and β-galactosidase activity, sugar, and product analyses.

Growth and β-galactosidase activity analyses were carried out as aforementioned. If needed, the cell dry weight was further measured according to the reported method (Ding et al., 2013). β-Glucosidase activity was evaluated by using p-nitrophenyl- β-D-gluco-pyranoside (pNPG) as described previously (Wang et al., 2013). The supernatant and cells of the resultant cultures were separated by centrifugation before the cells were washed twice with distilled water and finally re-suspended in distilled water. The supernatant and the re-suspended cells were tested for activity. Then the total activity and the ratio of extracellular activity to total activity were calculated.

Sugar and product analysis was carried out by HPLC (Waters Alliance 2695) with RI-detection after the separation on a guard column (Cation-H Refill Cartridges) and an Aminex HPX-87H column (Bio-Rad), using 4 mM H_2_SO_4_ as the mobile phase with a flow rate of 0.6 ml/min at 40°C.

### 2.5 RNA Extraction, Reverse Transcription, and PCR and qPCR Methods

The broth for RNA extraction was sampled and stored at −80°C as soon as possible. Then, the total RNA was extracted with a TIANDZ Column Fungal RNAout Kit (Beijing TIANDZ Gene Technology Co., Ltd.) according to the manufacturer’s instructions. Absorbance value determination, integrity analysis, reverse transcription reaction, and DNA pollution detection were all carried out according to the methods previously reported in our laboratory (Li et al., 2020).

The primers used for PCR and qPCR are described in Table 1. The product of the reverse transcription reaction was used for the amplification of the bands belonging to active/inactive *HAC1* (HAC1^i^ and HAC1^u^, respectively) with the pair of HAC1 primers (Table 1) (Kawazoe et al., 2017). Reactions were subjected to 30 PCR cycles of 95°C for 30 s, 54°C for 30 s and 72°C for 60 s. The PCR products were resolved on a 1.5% (w/v) agarose gel.

qPCR was carried out by using LightCycler 480 II and its corresponding software (Roche, Switzer-land) and ROCHE LightCycler@ 480 SYBR Green I Master and referring to the manual for specific operation steps. A total volume of 20 μl and 25 ng nucleic acid templates were used and the nucleic acid source was the RNA extraction or cDNA from RNA extraction. The qPCR conditions were as follows: 95°C for 5 min, 40 cycles of 95°C for 20 s, 55°C for 20 s, and 72°C for 20 s. The constitutive reference gene *ACT*1 and the 2^−△△CT^ method was utilized to normalize the amount of mRNA and obtain the relative expression level of the targeted genes. Each data point was referred to the control strain samples. The results represent the average and standard deviation of three independent biological replicates. Specially, all evaluating and analyzing experiments in aforementioned sections were repeated at least three times with consistent results.

## 3 Results

### 3.1 Amplification of the Donor DNA Fragment and Construction of Recombinant Indicating Strains and β-Glucosidase-Expressing Strains

In our previous work, we constructed the indicator strain W303-1A (*leu* 2::UPRE-*lac*Z, abbreviated as WZ) (Zhang et al., 2020) (Figure 1; Table 1), using the clustered regularly interspaced short palindromic repeats (CRISPR)-CRISPR associated protein nine (Cas9) method, with UPRE-*lac*Z as a reporter gene. Specifically, UPRE contains a 22 bp DNA sequence, 5′-GGAACTGGACAGCGTG TCGAAA-3′, and a 250 bp upstream sequence before the initial codon of the *CYC*1gene (Cox et al., 1993; Dallbey et al., 2009); and has *Lac*Z, a commonly used reporter (Cox et al., 1993). The 20 bp guide DNA sequence selected was 5′ TAT​TTA​CTT​TGG​TAA​GAG​AA 3′, corresponding to 423–442 nt of the *LEU*2 open reading frame (ORF), according to the design principle of the CRISPR-Cas9 method (DiCarlo et al., 2013). The PAM site sequence AGG, corresponding to 443–445 nt of the *LEU*2 ORF, was deleted and replaced by a UPRE-*lac*Z fragment in the genome of strain WZ. The resultant strain was selected by plating onto yeast potato dextrose (YPD) agar plates with tunicamycin and X-Gal. Tunicamycin induced the UPR, leading to the expression of *lac*Z, which enzyme catalyzes the substrate X-Gal to produce blue colonies. This phenotype indicated the existence and function of UPRE-*lac*Z fragment in cells.

In addition, we isolated and obtained the haploid strain K-a, derived from the diploid industrial yeast strain TH-AADY (Angel Yeast Co., Ltd., Yichang, China), and further proved that the CRISPR-Cas9 method was feasible in this strain (Lu et al., 2019). To conveniently design a guide sequence and integration site, auxotroph marker-encoding genes were sequenced, and the results proved that strain K-a contained the same DNA sequence of the *LEU2* ORF as that of the strain W303-1A. Therefore, the same strategy was carried out to construct the indicator strain KZ (Figure 1; Table 1; Section 2.2).

Furthermore, β-glucosidase-expressing strains were obtained by transforming β-glucosidase- Expressing plasmids BG and BG-cwp2 into strains WZ and KZ, respectively. As a control, the plasmid YEplac195 was also transformed. The results of the effect of secreting or displaying β-glucosidase using plasmids BG or BG-cwp2, respectively, has been reported previously (Wang et al., 2013; Ding et al., 2018).

### 3.2 Growth and UPR of Indicator Strains WZ and KZ in the Presence of Tunicamycin, Ethanol, and Acetic Acid

**FIGURE 2:**
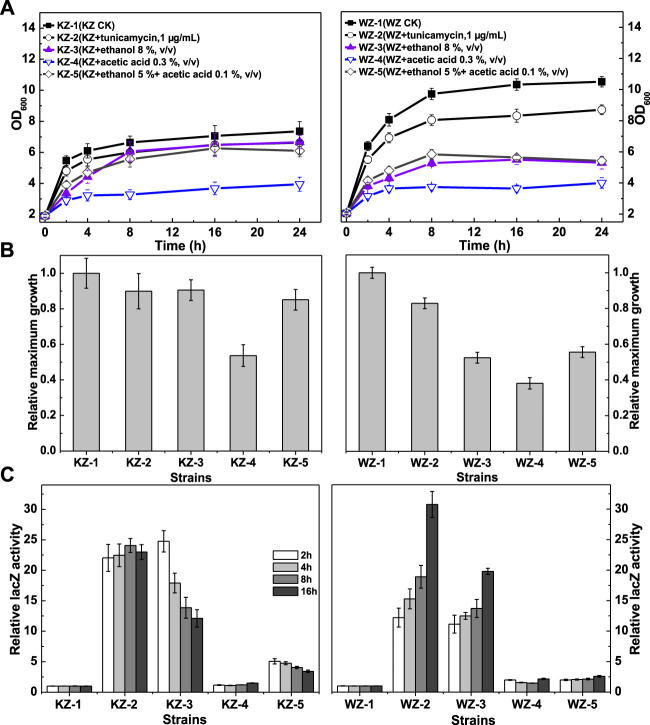
The growth and β-galactosidase activity of strains KZ and WZ in the YPD medium with different additives. **(A)** The growth curve; **(B)** the ratio of maximum OD_600_ value to that of controls KZ-1 or WZ-1; and **(C)** the ratio of β-galactosidase activity to that of controls KZ-1 or WZ-1. The original data was shown in Supplementary Material Table 1.

In YPD, no significant growth difference between the indicator strains and their host strains were observed (data not shown). Previous reports suggested that ethanol and acetic acid might induce a UPR during the fermentation process of cellobiose or lignocellulosic hydrolysates by the constructed β-glucosidase-expressing strains (Navarro-Tapia et al., 2016; Kawazoe et al., 2017; Navarro-Tapia et al., 2018; Cedras et al., 2019). Therefore, in the present study, ethanol and acetic acid, as well as tunicamycin as a control, were first selected to investigate the UPR in the indicator strains KZ and WZ. The growth data (Figures 2A,B) indicated that while the maximum OD_600_ value of KZ-1 was significantly lower than that of WZ-1, different additives inhibited growth by variable degrees. Acetic acid induced the highest growth inhibition and tunicamycin induced the lowest. In addition, the growth of strain KZ was inhibited to a lower extent than that of strain WZ, which implied that strain KZ has higher ethanol and acetic acid tolerance compared to strain WZ, which is in agreement with previous observations of the parental strains K-a and W303-1A (data unpublished).

The β-galactosidase activity data (Figure 2C) indicated that the UPR varied greatly according to the strains and additives, but did not seem to be related to growth. From high to low values, the β-galactosidase activity in the two strains in the presence of the additives was: tunicamycin > 8% ethanol (v/v) >5% ethanol (v/v) + 0.1% acetic acid (v/v) > 0.3% acetic acid (v/v). The ratio values of the latter two additives were 5.07–3.42 and 1.10–1.49 for strain KZ, and 1.99–2.57 and 1.46–2.15 for strain WZ, respectively. Thus, acetic acid showed only a slight activating effect.

In strain KZ, the β-galactosidase activity increased rapidly, peaked at about 2 h, and then decreased quickly. By contrast, the enzyme activity in strain WZ increased slowly and peaked at 16 h.

Taken together, these results demonstrated obvious differences in the rate and degree of the UPR between the strains. As a control, the β-galactosidase activity values of strain KZ and WZ in YPD were (10.65 ± 0.61)–(12.06 ± 0.59) and (22.2 ± 1.09)–(24.1 ± 1.35) U/mg protein, respectively.

### 3.3 Aerobic Growth, β-Glucosidase Production, and the UPR of β-Glucosidase-Expressing Strains in 2% Cellobiose

**FIGURE 3:**
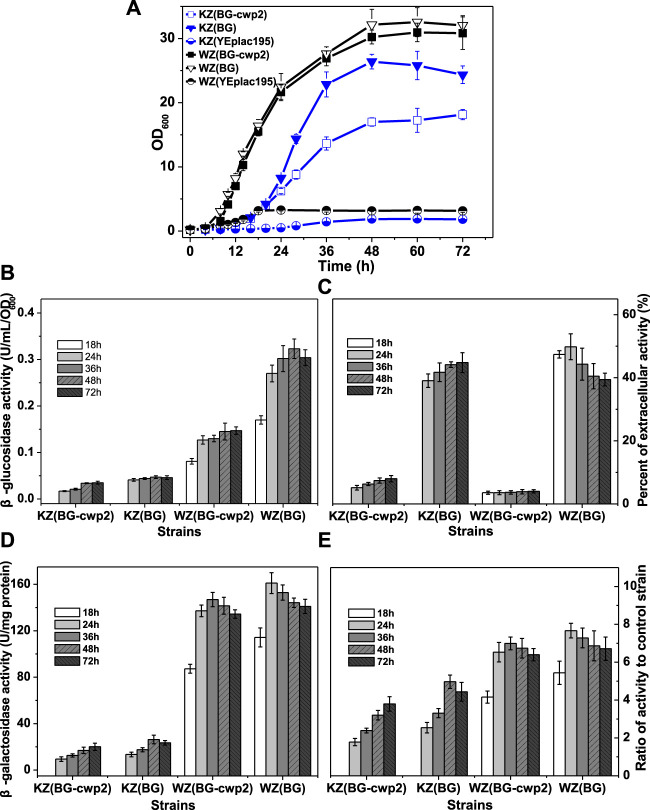
The growth, β-glucosidase, and β-galactosidase activities of the recombinant strains aerobically in 2% cellobiose. **(A)** The growth curve; **(B)** total β-glucosidase activity (U/ml/OD_600_), not detected for control strains KZ (YEplac195) and WZ (YEplac195); **(C)** the percent of extracellular β-glucosidase activity (%); **(D)** the β-galactosidase activity (U/mg protein); and **(E)** the ratio of β-galactosidase activity value of four strains to that of control strain KZ (YEplac195) and WZ (YEplac195), 5.30 ± 0.83 and 21.0 ± 0.95 U/mg protein, respectively. The original data was shown in Supplementary Material Table 2.

The catalytic activity of β-glucosidase toward cellobiose allows recombinant strains that express β-glucosidase to utilize cellobiose directly for growth and ethanol production. Here, 2% cellobiose medium, YPC, was first selected, and the aerobic growth, β-glucosidase, and β-galactosidase production of the recombinant strains were determined (Figure 3). As expected, the growth rates of strain WZ (BG-cwp2) and WZ (BG) were similar (Ding et al., 2018); however, the growth of strains KZ (BG-cwp2) and KZ (BG) differed (Figure 3A). Their OD_600_ values at 48 h, which were close to the maximum during the whole period, were 30.23 ± 1.08 WZ (BG-cwp2), 32.15 ± 1.41 WZ (BG), 17.00 ± 0.65 KZ (BG-cwp2), and 26.40 ± 1.14 KZ (BG), respectively.

Growth in the YPC medium depends on β-glucosidase production; therefore, the growth rate reflects the β-glucosidase production. Figure 3B shows that the total β-glucosidase activities of strains KZ (BG-cwp2) and KZ (BG) were also far lower than those of WZ (BG-cwp2) and WZ (BG), and their maximum activities were 0.035 ± 0.002 KZ (BG-cwp2), 0.047 ± 0.003 KZ (BG), 0.147 ± 0.008 WZ (BG-cwp2), and 0.323 ± 0.021 WZ (BG) U/ml/OD_600_. Interestingly, the ratios of β-glucosidase activity of KZ (BG) to that of KZ (BG-cwp2) and WZ (BG) to that of WZ (BG-cwp2), were relatively stable. The differences in the ratios of extracellular activity between strains KZ (BG) and WZ (BG), or between KZ (BG-cwp2) and WZ (BG-cwp2), were also not significant, at (39.08 ± 2.15) %—(49.82 ± 2.11) % and (3.55 ± 0.21) %—(8.00 ± 0.37) %, respectively (Figure 3C).

Furthermore, the β-galactosidase activities of the four strains correlated positively with their corresponding β-glucosidase values; however, the relationship was not linear (Figures 3B,D). All the β-galactosidase values of strain KZ-based samples were much lower than those of strain WZ-based samples (Figure 3D). However, when the activity data were expressed as ratios to that of the control, and their difference was not significant (Figure 3E). The maximum ratio values of strain KZ (BG-cwp2), KZ (BG), WZ (BG-cwp2), and WZ (BG) were 3.79, 4.97, 6.99, and 7.67, respectively. This clearly showed that plasmid BG led to higher β-galactosidase as well as β-glucosidase activities than plasmid BG-cwp2 in both hosts. In addition, plasmid BG allowed the cells to reach the maximum value of β-galactosidase activity more quickly than did plasmid BG-cwp2.

HPLC analysis revealed that ethanol and acetic acid were produced, with maximum titers of 5.38–6.09 g/L and ≤0.302 g/L, respectively. According to the results from Section 3.2, we suspected that ethanol or acetic acid did not contribute to the UPR, because their concentrations in the fermentation culture were too low.

### 3.4 Oxygen-Limited Growth, β-Glucosidase and Ethanol Production, and the UPR of β-Glucosidase-Expressing Strains in 10% Cellobiose

**FIGURE 4:**
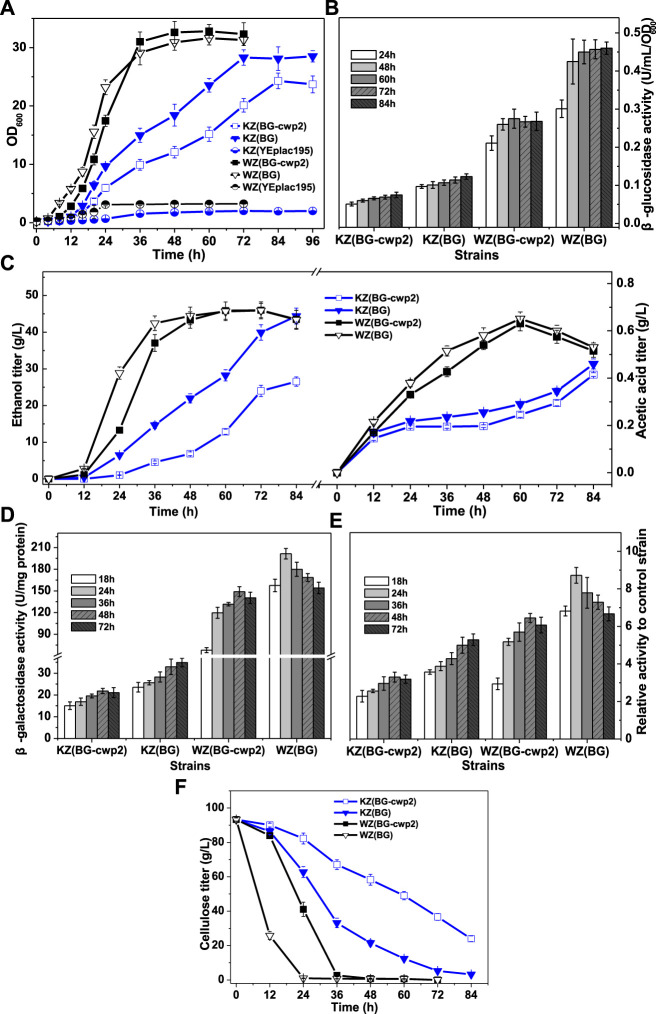
The growth, sugar consumption, β-glucosidase, ethanol and acid production, and β-galactosidase activity of the recombinant strains anaerobically in 10% cellobiose. **(A)** The growth curve; **(B)** total β-glucosidase activity (U/ml/OD_600_), not detected for control strains WZ (YEplac195) and KZ (YEplac195); **(C)** ethanol and acetic acid production; **(D)** the β-galactosidase activity (U/mg protein); **(E)** the ratio of β-galactosidase activity value of four strains to that of control strains KZ (YEplac195) and WZ (YEplac195), 6.60 ± 0.85 and 23.1 ± 0.93 U/mg protein, respectively; and **(F)** the cellobiose curve. The original data was shown in Supplementary Material Table 3.

Oxygen conditions greatly influences enzyme expression levels, cell growth, and fermentation performance. In addition, a high sugar concentration will lead to a high production of ethanol and other by-products. To observe the possible mutual interaction of UPR activation with exogenous gene expression, ethanol, and other by-products, we evaluated oxygen-limited growth, β-glucosidase activity, ethanol production, and β-galactosidase activity of the recombinant strains in media containing 10% cellobiose. The results are summarized in Figure 4, which indicated a significant effect of the strain source and displaying or secreting expression mode of the exogenous gene.

First, the results showed that strains WZ (BG-cwp2) and WZ (BG) could quickly produce sufficient β-glucosidase to utilize high cellobiose for growth and fermentation into ethanol and acetic acid (Figures 4A–C). In fact, the initial cellobiose concentration in media was measured by HPLC to be 93.3 ± 0.9 g/L. Strain WZ (BG-cwp2) and WZ (BG) exhausted cellobiose during 48–60 h and 24–36 h, respectively (Figure 4F). The maximum ethanol titer and yield reached 45.98 ± 2.31 g/L (5.495 ± 0.276%, v/v) and 91.7% (at 72 h) in WZ (BG-cwp2), and 45.91 ± 1.96 g/L (5.486 ± 0.234%, v/v) (72 h) and 91.5% in WZ (BG) (expressed as a percentage of the theoretical yield) (Figure 4C). At the same time, acetic acid was produced and reached the highest level of 0.631 g/L or 0.058% (v/v) in WZ (BG-cwp2), and 0.651 g/L or 0.060% (v/v) at 60 h in WZ (BG) (Figure 4C).

By contrast, the results showed that strains KZ (BG-cwp2) and KZ (BG) utilized cellobiose but grew slowly (Figures 4A–C,F), possibly because of insufficient enzyme supply (Figure 4B). In detail, their maximum values of OD_600_ reached 24.30 ± 1.34 in KZ (BG-cwp2) and 28.30 ± 1.32 in KZ (BG) at 84 and 72 h, respectively (Figure 4A). The cellobiose, ethanol, and acetic acid concentrations of strain KZ (BG-cwp2) at 84 h were 23.96 ± 1.67, 26.55 ± 1.26, and 0.415 ± 0.008 g/L, respectively. Those of strain KZ (BG) at 84 h were 3.21 ± 0.28,44.41 ± 2.16, and 0.460 ± 0.021 g/L, respectively (Figures 4C,F).

In our experience, a higher cellobiose content resulted in a higher β-glucosidase production level. In fact, the maximum values of total β-glucosidase activity from the four strains, KZ (BG-cwp2), KZ (BG), WZ (BG-cwp2), and WZ (BG), were 0.075 ± 0.007, 0.123 ± 0.006, 0.275 ± 0.015, and 0.460 ± 0.036 U/ml/OD_600_, respectively (Figure 4B). Compared with those in 2% cellobiose media (Figure 3B), the maximum values increased by 2.14, 2.62, 1.87, and 1.42 times in 10% cellobiose media in the four strains, respectively (Figure 4B).

In 10% cellobiose, there was also a positive, but not linear, relationship between the β-glucosidase and β-galactosidase activities (Figures 4B,D,E). Similar to that in 2% cellobiose, the β-galactosidase values from strain KZ-based samples were lower than those of strain WZ-based samples (Figures 4D,E). The maximal ratio values of β-galactosidase activity of strain KZ (BG-cwp2), KZ (BG), WZ (BG-cwp2), and WZ (BG) became 3.30, 5.29, 6.45, and 8.72, respectively (Figure 4E). Therefore, the results suggested that anaerobic fermentation with 10% cellobiose increased the difference in β-galactosidase activity levels between strains KZ (BG-cwp2) and KZ (BG), or WZ (BG -cwp2) and WZ (BG). Additionally, strain WZ (BG) reached the maximum value of β-galactosidase activity the most quickly in both 2 and 10% cellobiose, at 24 and 48 h, respectively, after which, the values decreased.

The times taken by the three strains, WZ (BG), WZ (BG-cwp2), and KZ (BG) to produce ≥5% (v/v) ethanol, which is equal to 41.85 g/L ethanol, were 36, 48, and 84 h, respectively. At those times, the ethanol titers in the three strains were 5.07, 5.18, and 5.31% (v/v), respectively, and the acetic acid titers were 0.515, 0.540, and 0.460 g/L, corresponding to 0.047, 0.050, and 0.042% (v/v), respectively. Such ethanol and acetic acid titers were close to those of the group treated with 5% (v/v) ethanol +0.1% (v/v) acetic acid in Section 3.2 (Figure 2) and would probably make a contribution to UPR signaling. It would be a challenge to determine the effect of ethanol or acetic acid on the UPR, because their titers varied with the course of fermentation, especially when the UPR had been induced by other factors, for example, exogenous protein expression.

### 3.5 Quantitative PCR of UPR Target Genes and *HAC1* mRNA Analysis

UPRE contains a 22 bp cis-acting element, that is, necessary and sufficient for the induction of the yeast *KAR2* (BiP) gene in response to unfolded proteins (Dallbey et al., 2009). In fact, UPRE-*lac*Z has often been used in UPR studies as a reporter gene (Dallbey et al., 2009; Navarro-Tapia et al., 2018). It has also been proven to respond to tunicamycin, ethanol, or acetic acid (Figure 2), in addition to β-glucosidase production (Figure 3). Therefore, it could provide a direct and quantitative comparison of UPR signaling from different inducers.

**FIGURE 5:**
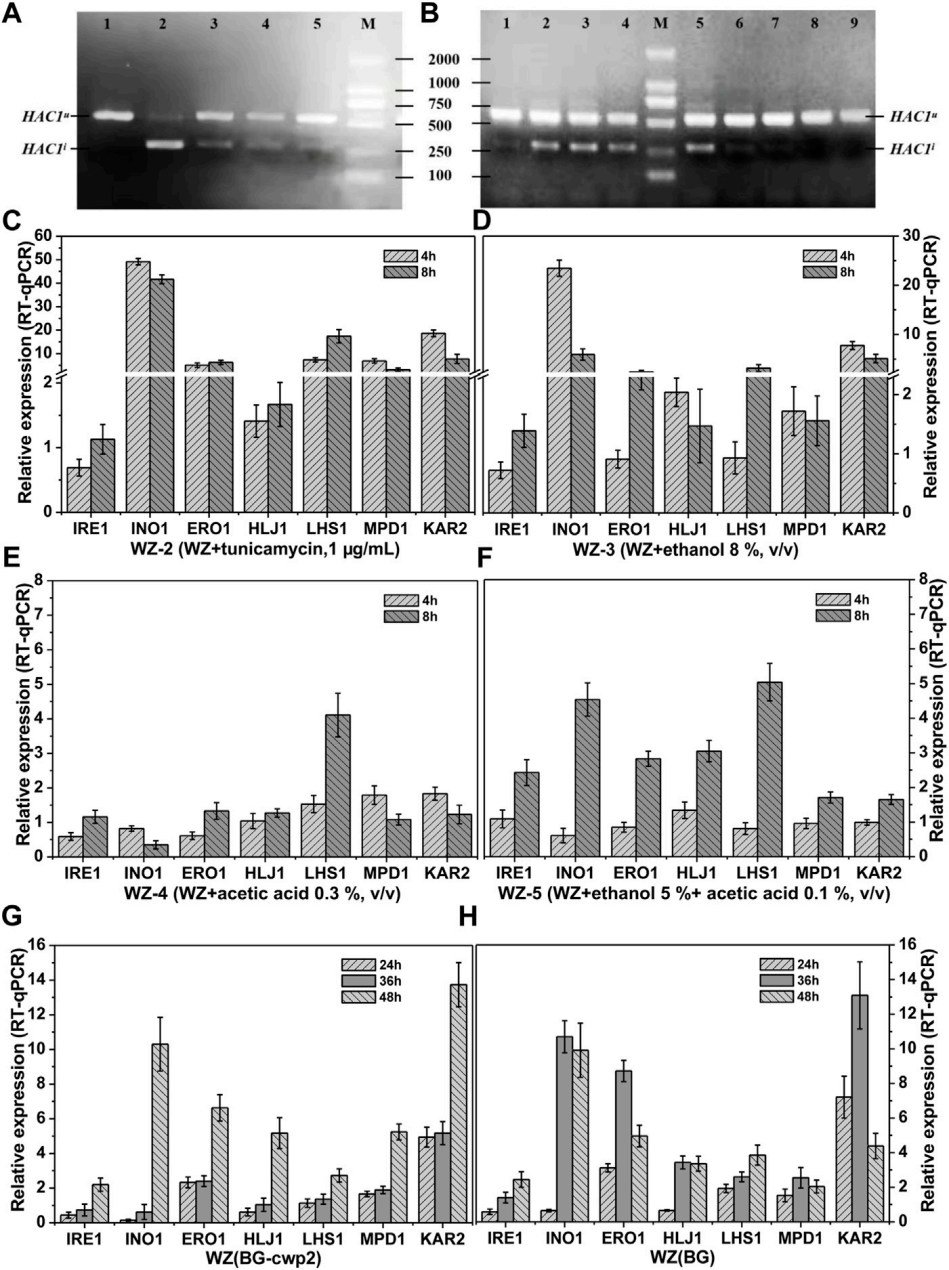
*HAC1* mRNA splicing and relative expression of UPR target genes. *HAC1* mRNA splicing **(A)** and the relative expression level of UPR target genes **(C–F)** of strains WZ in YPD medium with different additives; *HAC1* mRNA splicing **(B)** and relative expression level of UPR target genes **(G–H)** of strains WZ (BG-cwp2) and WZ (BG) in 10% cellobiose. **(A)** lanes 1∼5, WZ-1∼WZ-5, 8 h; **(B)** lanes 1,8 and 9, 24, 36 and 48 h fermentation time of strain WZ (YEplac195); lanes 2∼4, 24, 36 and 48 h fermentation time of strain WZ (BG); lanes 5∼7, 24, 36 and 48 h fermentation time of strain WZ (BG-cwp2). Each data point was referred to the control strain samples, and the mRNA levels of the target genes were determined by qPCR after normalization with constitutive control *ACT1* gene. The results represent the average and standard deviation of three independent biological replicates. The original data was shown in Supplementary Material Table 4.

To investigate the effect and mechanism on the UPR of the abovementioned inducers, it is necessary to carry out molecular detection. The canonical UPR pathway genes in yeast cells include those encoding an ER membrane sensor, IreIp, a transcription factor, HacIp, and Kar2p. When activated by the accumulation of aberrant folded proteins in the ER, IreIp catalyzes the splicing of *HAC1* to activate hundreds of genes that restore the normal ER function. Kar2p acts as a chaperone to mediate protein folding in the ER and regulates the UPR *via* interaction with Ire1p (Dallbey et al., 2009). While no protein de-naturation has been described at 6% or 8% ethanol, ethanol was reported to activate *INO1* gene expression and further enhance the UPR by membrane fluidification (Navarro-Tapia et al., 2016; Navarro-Tapia et al., 2018). *INO1* encodes an essential enzyme for inositol biosynthesis and is activated to restore lipid levels (Navarro-Tapia et al., 2018). Acetic acid was demonstrated to lead to the accumulation of misfolded proteins in the ER and the activation of Ire1p and Hac1p (Kawazoe et al., 2017). Therefore, in the present study, *HAC1* mRNA splicing and the expression of eight genes (Table 1) were analyzed by RNA extraction from the samples stored at −80°C in Sections 3.2–3.4 experiments. An additional four genes involved in protein folding, *ERO1*, *LHS1*, *HLJ1*, and *MPD1* were assessed (Dallbey et al., 2009; Navarro-Tapia et al., 2018). The results are shown in Figure 5.

As expected, spliced *HAC1* mRNA was shown to accumulate by varying degrees, which correlated positively with the corresponding β-galactosidase values (Figures 2C, 4D, 5A,B). In fact, the spliced *HAC1* mRNA accumulation in the samples of strains WZ and KZ with 0.3% (v/v) acetic acid or 5% ethanol +0.1% acetic acid (v/v) was slight (Figure 5A and data not shown). This result was different from a previous report (Kawazoe et al., 2017). We hypothesized that the main causes were the differences in the strains, medium, and the initial OD_600_ value when exposed to stress. A similar phenomenon was also observed for strain KZ in the fermentation broth, especially in the presence of 2% cellobiose (data not shown).

The results in Figures 5C–F demonstrated the characteristic responses to the different inducers, which generally agreed with changes observed in previous articles (Navarro-Tapia et al., 2016; Kawazoe et al., 2017; Navarro-Tapia et al., 2018). The existence of ethanol led to *INO1* to be among the most activated genes. The expression of *KAR2* was relatively high and constant, as expected; however, *IRE1* only showed a slightly upregulated expression. In addition, the response of *LHS1* appeared to be specific for acetic acid and had a higher expression level than the other genes (Figures 5C,D).

The results in Figures 5G–H indicated that the expression levels of *INO1* and *KAR2* were significantly activated and upregulated in cells in the fermentation broth with 10% cellobiose. The times to obtain the highest level of *INO1* expression were 36 and 48 h for strains WZ (BG) and WZ (BG-cwp2), respectively, and the corresponding ethanol titers were 5.07, and 5.18% (v/v), respectively (Figure 4). Comparing all of the data under 10% cellobiose (Figures 4, 5) with those under 2% cellobiose (Figures 2, 3), allowed us to hypothesize that ethanol also contributes to UPR signaling over a certain titer range. For acetic acid, the data did not reveal its effect in the UPR in the fermentation broth, possibly because its titer was too low.

## 4 Discussion

Although there has been a lot of research on protein quality control mechanisms and secretory recombinant protein production in yeast (Orlean and Menon, 2007; Pittet and Conzelmann, 2007; Dallbey et al., 2009; Young and Robinson, 2014; Bao et al., 2017; Cedras et al., 2019; Sun and Brodsky, 2019; Davison et al., 2020; Thak et al., 2020), this study provides the first evaluation and direct comparison of the UPR during cellobiose utilization of recombinant haploid yeast derived from Angel and W303-1A strains expressing β-glucosidase under the same conditions. On the basis of quantifying UPR induction, we attempted to analyze all possible factors that contribute to UPR signaling in a specific fermentation system, and further investigated the interconnectedness and mutual influence on UPR among the inducers.

Although *S. cerevisiae* is reported to naturally have a relatively low secretory pathway capacity (Bao et al., 2017), there is still a significant difference in secretion capacity between different strains (Davison et al., 2020)**,** which should be evaluated and exploited. Angel Yeast is widely used in large-scale industrial bio-ethanol production in China and is also among the world-famous industrial yeast brands (http://www.angelyeast.com) (Zou et al., 2021), being an ideal candidate for lignocellulose conversion. However, Angel Yeast TH-AADY-derived haploid strains were repeatedly found to have much lower cellulase enzyme activity compared to the host strain W303-1A (Wang et al., 2013; Hong et al., 2014; Ding et al., 2018; Zou et al., 2021).

Therefore, one aim of this study was determining why the cellulase activity produced by Angel-derived strains is low, how it relates to UPR induction, and whether the UPR capacity is a limiting factor. The results of growth and UPR response evaluations of strains KZ and WZ in tunicamycin, ethanol, and acetic acid proved that strain KZ has a stronger resistance to those additives, and could more quickly activate and then inactivate the UPR (Figure 2C). By contrast, the results in 2% and 10% cellobiose both showed poorer β-glucosidase production and lower UPR signaling in KZ-based strains than in WZ-based strains, which led to a slower growth and a lower maximum OD_600_ value (Figures 3, 4). The observed positive correlation between the two enzyme activities possibly implied a low processing capacity of the secretory pathway in the parental strain K-a, which implied a low threshold to activate the UPR to degrade unfolding proteins.

The second aim of this study was to observe how the displaying and secreting pattern of heterologous protein expression influenced the UPR and further determine if there is a relationship between the UPR and the metabolic burden, which has been reported to be an extra by displaying over secreting (Ding et al., 2018). Cellobiose cannot be transported into yeast, thus its utilization depends on the production of secreted enzymes; however, we observed a non-linear relationship between enzyme expression and biomass synthesis and/or product formation based on substrate utilization. The maximum value of β-glucosidase activity in WZ (BG) cells was 2.20 and 1.68 times the level of WZ (BG-cwp2) cells in 2% and 10% cellobiose, respectively (Figures 3B, 4B). However, their growth and fermentation rates were very similar (Figures 3A, 4A,C). Herein, the metabolic burden induced by β-glucosidase displaying over secreting seems to be low for the WZ-based strains. By contrast, while the maximum values of β-glucosidase activity in KZ (BG) cells were 1.35 and 1.64 times the level of KZ (BG-cwp2) cells in 2 and 10% cellobiose, respectively (Figures 3B, 4B). There was a marked difference in their growth and fermentation rates (Figures 3A, 4A,C), which demonstrated the existence of a significant metabolic burden, as reported previously (Ding et al., 2018).

Unexpectedly, only strains KZ (BG) and KZ (BG-cwp2) showed that the β-galactosidase activity was essentially proportional to the β-glucosidase activity, or were at least positively correlated. In other words, the UPR seems to have no direct relation to displaying or secreting, regardless of the oxygen supply or cellobiose concentration. However, this was not the case for strains WZ (BG) and WZ (BG-cwp2). Compared to the difference in β-glucosidase activity between strains WZ (BG-cwp2) and WZ (BG), the β-galactosidase activity of strain WZ (BG-cwp2) was similar to that of strain WZ (BG), and seemed to be abnormally high (Figures 3D,E, 4D,E).

It is necessary to first analyze which factors induce UPR signaling. According to previous reports (Dallbey et al., 2009; Young and Robinson, 2014; Navarro-Tapia et al., 2016; Kawazoe et al., 2017; Navarro-Tapia et al., 2018; Cedras et al., 2019; Sun and Brodsky, 2019), it was speculated that there are at least three factors that activate the UPR in the cellobiose utilization system in this study, heterologous β-glucosidase expression, ethanol, and acetic acid production. However, only the combination of mild acetic acid stress (0.1% acetic acid) and mild ethanol stress (5% ethanol) was reported to induce the UPR, whereas neither mild ethanol stress nor mild acetic acid stress individually activated the UPR (Kawazoe et al., 2017). 6 or 8 % ethanol was observed to activate UPR but did not de-nature proteins (Navarro-Tapia et al., 2018). By contrast, a minimal ethanol titer of 4%–5% v/v is required to render distillation economically viable; however, ethanol concentrations in lignocellulose fermentations struggle to reach these concentrations (Brandt et al., 2021). Therefore, in this study, oxygen-limited 10% cellobiose fermentation was designed and ≥5% v/v maximum ethanol titers were obtained at the later stage of fermentation (Figure 4C).

In such a system, the switch from β-glucosidase expression to substrate utilization, and then biomass synthesis and by-product production, is supposed to influence the dynamics of the UPR. To detect the mRNA levels of both the UPR central components and UPR target genes, would help to reveal UPR inducers and response dynamics. Comparisons of the curves of enzyme activity, growth, substrate, and products in 2 and 10% cellobiose (Figures 3, 4), and the relative expression level of different genes (Figure 5), made it reasonable to conclude that the constitutive heterologous expression would result in a durable UPR induction, and ethanol or acetic acid activation depends on the titer produced. In 10% cellobiose fermentation, the ethanol titer was up to 5% (v/v) and could induce high expression of the specific target gene *INO1* and should lead to UPR signaling. Regrettably, detection and analysis still could not differentiate and quantify the contribution of ethanol to UPR signaling in such a complex fermentation system. Such an analysis requires multi-faceted investigations.

An important issue is how and why the display and secretion pattern of β-glucosidase expression influenced UPR activation, especially in strain WZ cells. Undoubtedly, the key step is glycosylphosphatidylinositol (GPI) anchor addition, an essential maturation process of secretory proteins in the ER, which is also the first step to distinguish displaying from secreting of secretory proteins (Orlean and Menon, 2007; Pittet and Conzelmann, 2007). GPI lipids are synthesized in the ER and added onto proteins by a pathway comprising 12 steps, carried out by 23 gene products, 19 of which are essential (Pittet and Conzelmann, 2007). Thus, GPI anchoring of cell-surface proteins is the most complex and metabolically expensive lipid post-translational modification described to date (Orlean and Menon, 2007).

Consequently, the growth defect observed in this study was expected and was also similar to that reported previously (Ding et al., 2018; Brandt et al., 2021). The phenomenon that the defect was more serious in strain KZ (BG-cwp2) cells than that in strain WZ (BG-cwp2) cells supported the speculation that the growth defect stems from a combination of relatively low activity on cellobiose and the metabolic burden imposed by enzyme expression. In addition, anchoring moieties contribute to the growth difference of the cell-surface-display-expressing strains under both aerobic and anaerobic conditions (Ding et al., 2018).

The results of the present study allowed us to cautiously speculate that the GPI anchoring process by an anchored peptide-encoding sequence (cwp2) in plasmid BG-cwp2 would bring about two results: 1) An increased expression of endogenous anchoring-related genes inside cells, which would boost UPR induction and make the resultant signal comparable to that during the secretion of β-glucosidase; 2) extra material and energy consumption, which would lead to biosynthesis stress and this lagged behind β-glucosidase expression in cells and cellobiose hydrolysis. Both of these pathways imposed a metabolic burden.

The maximum values of the ratio of β-galactosidase activity in aerobic 2% cellobiose culture were 3.79, 4.97, 6.99, and 7.67 for the four strains KZ (BG-cwp2), KZ (BG), WZ (BG-cwp2), and WZ (BG), respectively; whereas, those values in anaerobic 10% cellobiose culture changed to 3.30, 5.29, 6.45, and 8.72, respectively. Although β-glucosidase activity by all strains increased under anaerobic 10% cellobiose (Figure 5), it was still apparent that the difference in the ratio of β-galactosidase activity between strains KZ (BG-cwp2) and KZ (BG), and WZ (BG-cwp2) and WZ (BG), increased. One reason behind this might be attributed to biomass synthesis. The biomass yield (expressed as g of dry cell weight (DCW) /g sugar consumed) in anaerobic 10% cellobiose was determined to be only from one-third to one-fifth of the value under aerobic 2% cellobiose conditions (data not shown). This would greatly alleviate the aforementioned biosynthesis stress from GPI anchoring and also decrease UPR induction in KZ (BG-cwp2) or WZ (BG-cwp2) cells.

Therefore, the results of the present study indicated a mutual interaction of the UPR response and the metabolic burden that varied according to the host strain, protein expression level, displaying or secreting mode, and fermentation conditions. Undoubtedly, the host strain had the most significant effect. Here, the comparison of W303-1A- and K-a-derived strain demonstrated the relationship between secretory recombinant protein production and the UPR and metabolic burden. Low β-glucosidase production from KZ-derived strains should result in UPR and/or ERAD induction (Davison et al., 2020), and in turn, a low induction signal reflects a limited ER processing capacity in cells. Expensive displaying exacerbates the metabolic burden resulting from insufficient or slow enzymes and glucose substrate supply. By contrast, host strain W303-1A confers on its derived strains excellent enzyme productivity and secretory pathway capacity, and a subtle difference in the metabolic burden of displaying over secretion.

A key challenge for bioengineering or synthetic biology is to balance different traits for a specific application. It is apparent that strain W303-1A is more suitable for use as a host to produce secretory recombinant proteins than strain K-a. When it is necessary to express heterologous genes in strain K-a, to broaden its substrate range to take full advantage of its industrial traits for ethanol production, it will be necessary to first enhance the UPR and ERAD activity and modulate its secretory pathway capacity. In fact, recently, much progress has been made in strain K-a modification based on the results of this study in our laboratory.

In summary, this study illustrates the differences in resistance to ethanol or acetic acid and the UPR between indicator strains KZ and WZ. The results of cellobiose utilization assays demonstrated the interaction of the UPR and the metabolic burden according to the strain source, anchoring moiety, oxygen supply, and cellobiose concentration. The OD_600_ values and β-glucosidase and β-galactosidase activities were shown to correlate positively with each other; however, these values in the KZ-derived strains were far lower than those in the WZ-derived strains under the conditions tested. Meanwhile, the metabolic burden induced by displaying over secreting was also much more serious in strain KZ than in strain WZ. Comparisons of the results under two different conditions implied that β-glucosidase expression would provide a durable inducing effect on the UPR, whereas, the effect of ethanol and acetic acid depends on the titer produced, and could be identified by detecting the expression levels of key specifically targeted genes. The results indicated that host strain W303-1A is a better secretory protein producer. Moreover, the first step to modify strain K-a for cellulosic ethanol fermentation would to break the bottleneck of the UPR capacity.

## Data Availability

The original contributions presented in this study are included in the article/Supplementary Material, further inquiries can be directed to the corresponding authors.
